# Crystal structure of tricarbon­yl[η^4^-6-*exo*-(tri­phenyl­phosphino)cyclo­hepta-2,4-dien-1-one]iron(0) tetra­fluoro­borate

**DOI:** 10.1107/S2056989024005747

**Published:** 2024-06-18

**Authors:** Kelsey C. Wong, Eric W. Reinheimer, Chip Nataro, Daniel R. Griffith

**Affiliations:** a Department of Chemistry Lafayette College, Hugel Science Center Easton PA 18042-1768 USA; b Rigaku Americas Corporation, 9009 New Trails Dr The Woodlands TX 77381 USA; University of Buenos Aires, Argentina

**Keywords:** crystal structure, phosphine, undergraduate, iron carbon­yl, piano stool, η^4^-cyclo­hepta-2,4-dien-1-one

## Abstract

The mol­ecular structure of tricarbon­yl[η^4^-6-*exo*-(tri­phenyl­phosphino)cyclo­hepta-2,4-dien-1-one]iron(0) tetra­fluoro­borate di­chloro­methane hemisolvate, [Fe(C_28_H_22_O_4_)(CO)_3_]BF_4_·0.5CH_2_Cl_2_, as determined by single-crystal X-ray diffraction is reported. The two independent tricarbon­yl[η^4^-6-*exo*-(tri­phenyl­phosphino)cyclo­hepta-2,4-dien-1-one] iron(0) cations and their corresponding anions form dimers, which constitute the asymmetric unit of the structure parallel to the (100) plane. Solid-state stability within that asymmetric unit as well as between neighboring dimeric units is afforded by C—H⋯O and C—H⋯F hydrogen bonds and C—H⋯π and *Y*—*X*⋯π (*Y* = B, C; *X* = F, O) inter­actions, which yield diperiodic sheets and a three-dimensional extended network.

## Abstract 2

The crystal structure of tricarbon­yl[η^4^-6-*exo*-(tri­phenyl­phosphino)cyclo­hepta-2,4-dien-1-one]iron(0) tetra­fluoro­borate is described. The two independent tricarbon­yl[η^4^-6-*exo*-(tri­phenyl­phosphino)cyclo­hepta-2,4-dien-1-one] iron(0) cations and their corresponding anions form dimers, which constitute the asymmetric unit of the structure within the (100) plane.

## 1. Chemical context

**Figure 1:**
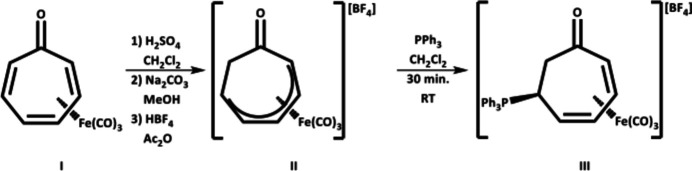
Tricarbon­yl(tropone)iron(0) (**I**), tricarbon­yl(η^5^-keto­cyclo­hepta­dien­yl)iron(II) tetra­fluoro­borate (**II**), and tricarbon­yl[η^4^-6-*exo*-(tri­phenyl­phosphino)cyclo­hepta-2,4-dien-1-one]iron(0) tetra­fluoro­borate (**III**) and the procedure outlining the synthesis of **III** from **I** and **II**.

This compound was prepared as part of a Course-based Undergraduate Research Experience (CURE) (Stone *et al.*, 2020 ▸; Huang *et al.*, 2019 ▸). The foundation of this CURE was to further examine addition reactions to tricarbon­yl(tropone)iron(0) (**I**) and tricarbon­yl(η^5^-keto­cyclo­hepta­dien­yl)iron(0) tetra­fluoro­borate (**II**) (Fig. 1 ▸). The research focus of one author lies in the synthesis of unique and diverse aza­polycyclic skeletons from common synthetic building blocks such as compound **I** due to the biological importance of such scaffolds. Although seven-membered carbocyclic rings are found in a number of biologically active natural products (Shoemaker & Griffith, 2021 ▸), their synthesis tends to present a greater challenge compared to similar five- or six-membered rings because of the increased enthalpic and entropic barriers associated with their formation (Phelan *et al.*, 2020 ▸; Huang *et al.*, 2018 ▸). The addition of a number of different nucleophiles to compound **II** has previously been reported, including amines (Phelan *et al.*, 2020 ▸), azide, and cyanide (Eisenstadt, 1975 ▸). This raised the question as to whether or not tri­phenyl­phosphine would be sufficiently nucleophilic to react with compound **II**. Previously, the reaction of several phosphines (PEt_3_, P^*n*^Pr_3_, P^*n*^Bu_3_ or PMe_2_Ph) with tricarbon­yl(η^5^-cyclo­hepta­dien­yl)iron(II) tetra­fluoro­borate in methyl­ene chloride resulted in the formation of the corresponding tri­carbon­yl[η^4^-(5-*exo*-phosphine)cyclo­hepta­diene]iron(0) tetra­fluoro­borate (Brown *et al.*, 1982 ▸). Similar to that system, the reaction of compound **II** and tri­phenyl­phosphine resulted in the formation of tricarbon­yl[η^4^-6-*exo*-(tri­phenyl­phosphino)cyclo­hepta-2,4-dien-1-one]iron(0) tetra­fluoro­borate (**III**) (Fig. 1 ▸). Ultimately, this and similar phospho­nium salts could be a precursor for Wittig olefinations that would provide efficient access to tropone rings with diverse substituents.
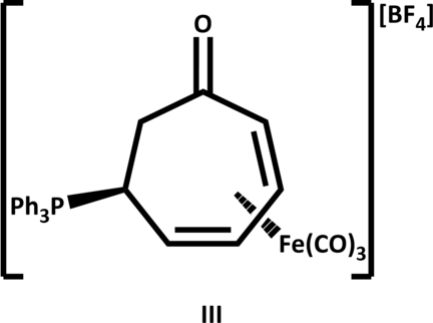


## 2. Structural commentary

**Figure 2:**
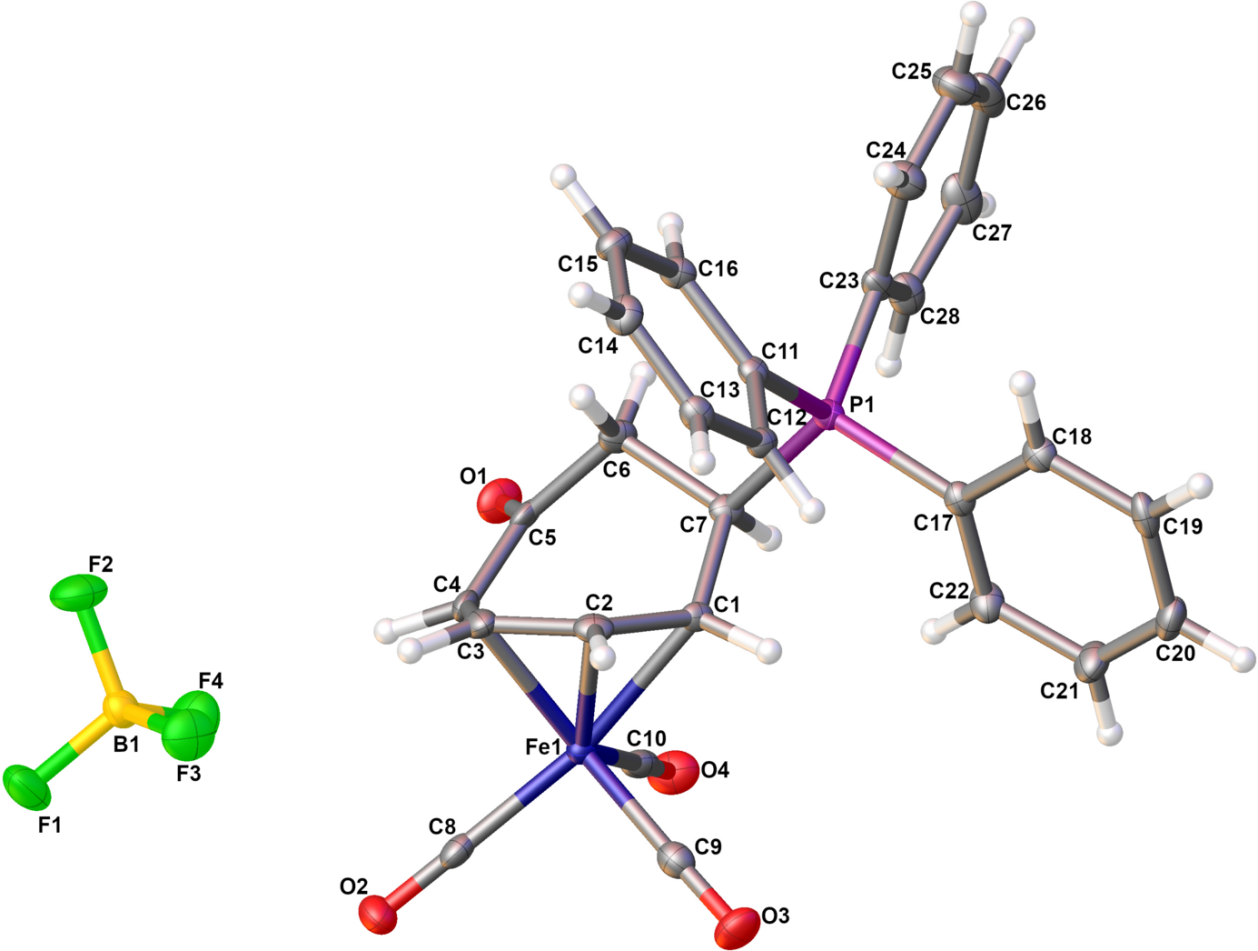
Single-crystal structure of one tricarbon­yl[η^4^-6-*exo*-(tri­phenyl­phosphino)cyclo­hepta-2,4-dien-1-one]iron(0) tetra­fluoro­borate (**III**) from the asymmetric unit with anisotropic displacement ellipsoids at the 50% probability level. The inter­stitial CH_2_Cl_2_ has been removed for the sake of clarity.

The single crystal X-ray structure of **III** crystallizes in the centrosymmetric triclinic space group *P*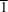 (Fig. 2 ▸). The asymmetric unit consists of two tricarbon­yl[η^4^-6-*exo*-(tri­phenyl­phosphino)cyclo­hepta-2,4-dien-1-one]iron(0) cations, two tetra­fluoro­borate anions (to balance the charge), and an inter­stitial CH_2_Cl_2_ solvent mol­ecule lying in solvent-accessible voids of ∼101 Å^3^. The iron tricarbonyl moieties adopt piano stool orientations with the cyclo­hepta-2,4-dien-1-one group (Fig. 2 ▸). Closer analysis of the thermal parameters of the [BF_4_]^−^ anions and CH_2_Cl_2_ solvent mol­ecule within the asymmetric unit showed no qualitative evidence of disorder.

## 3. Supra­molecular features

**Figure 3:**
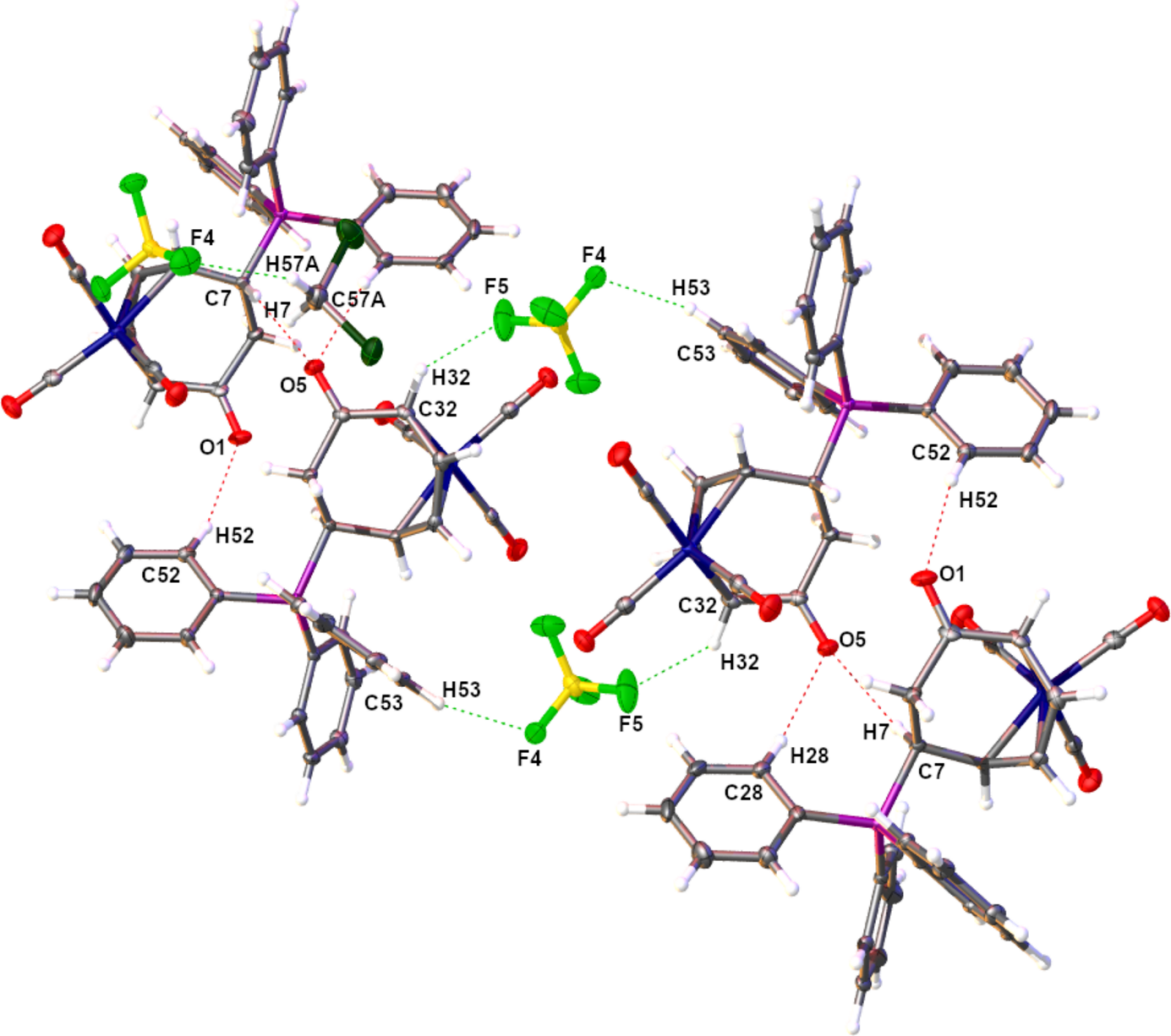
View of the C—H⋯O and C—H⋯F hydrogen bonds from the (101) plane of **III**. When coupled with the C—H⋯π and Y—*X*⋯π (*Y* = B, C; *X* = F, O) inter­actions, this repeat unit extends into a three-dimensional network. Anisotropic displacement ellipsoids have been set to the 50% probability level.

**Figure 5:**
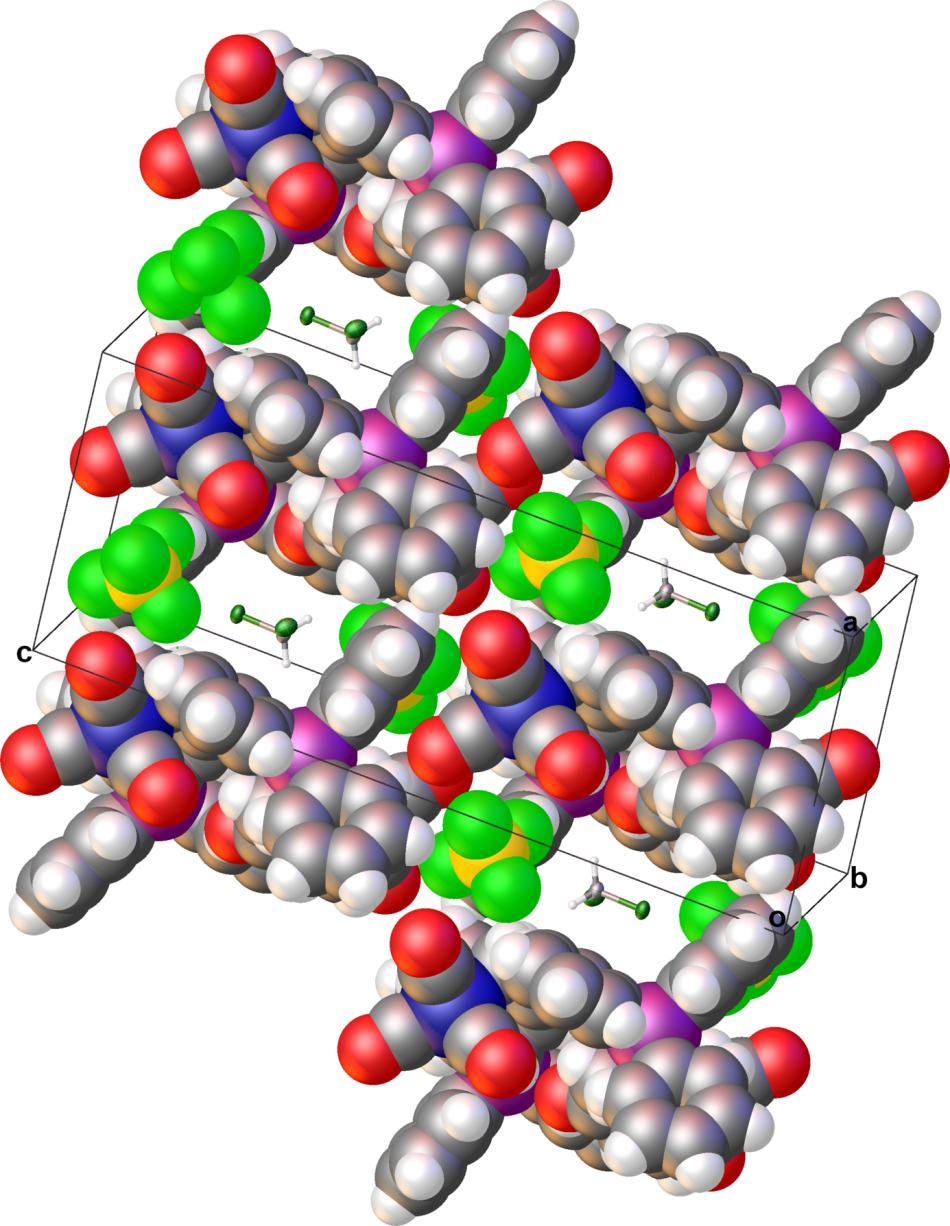
View into the (120) plane showing the inter­stitial CH_2_Cl_2_ solvent mol­ecules lying within the solvent-accessible voids of **III**. These voids are generated from the packing supported by the C—H⋯O and C—H⋯F hydrogen bonds and C—H⋯π and Y—*X*⋯π (*Y* = B, C; *X* = F, O) inter­actions. The anisotropic displacement ellipsoids for CH_2_Cl_2_ have been set to the 50% probability level.

Solid-state stability between the mol­ecules of **III** within the asymmetric unit is afforded by an array of C—H⋯O and C—H⋯F hydrogen bonds (Table 1 ▸) as determined through *PLATON* analysis (Spek, 2020 ▸). The two independent tri­carbon­yl[η^4^-6-*exo*-(tri­phenyl­phosphino)cyclo­hepta-2,4-dien-1-one]iron(0) cations from the asymmetric unit lie parallel to the (100) plane and are stabilized by inter­molecular C—H⋯O hydrogen bonds. The addition of C—H⋯F hydrogen bonding involving the [BF_4_]^−^ anions increases the dimensionality of the solid-state structure into both diperiodic sheets and extended 3D networks, which also contain C—H⋯π and *Y*—*X*⋯π (*Y* = B,C; *X* = F, O) inter­actions (Table 2 ▸) according to *PLATON* (Spek, 2020 ▸) (Figs. 3 ▸ and 4 ▸). The resulting 3D network was also found to contain solvent-accessible voids of ∼101 Å^3^ within which the inter­stitial CH_2_Cl_2_ was located (Fig. 5 ▸).

**Figure 6:**
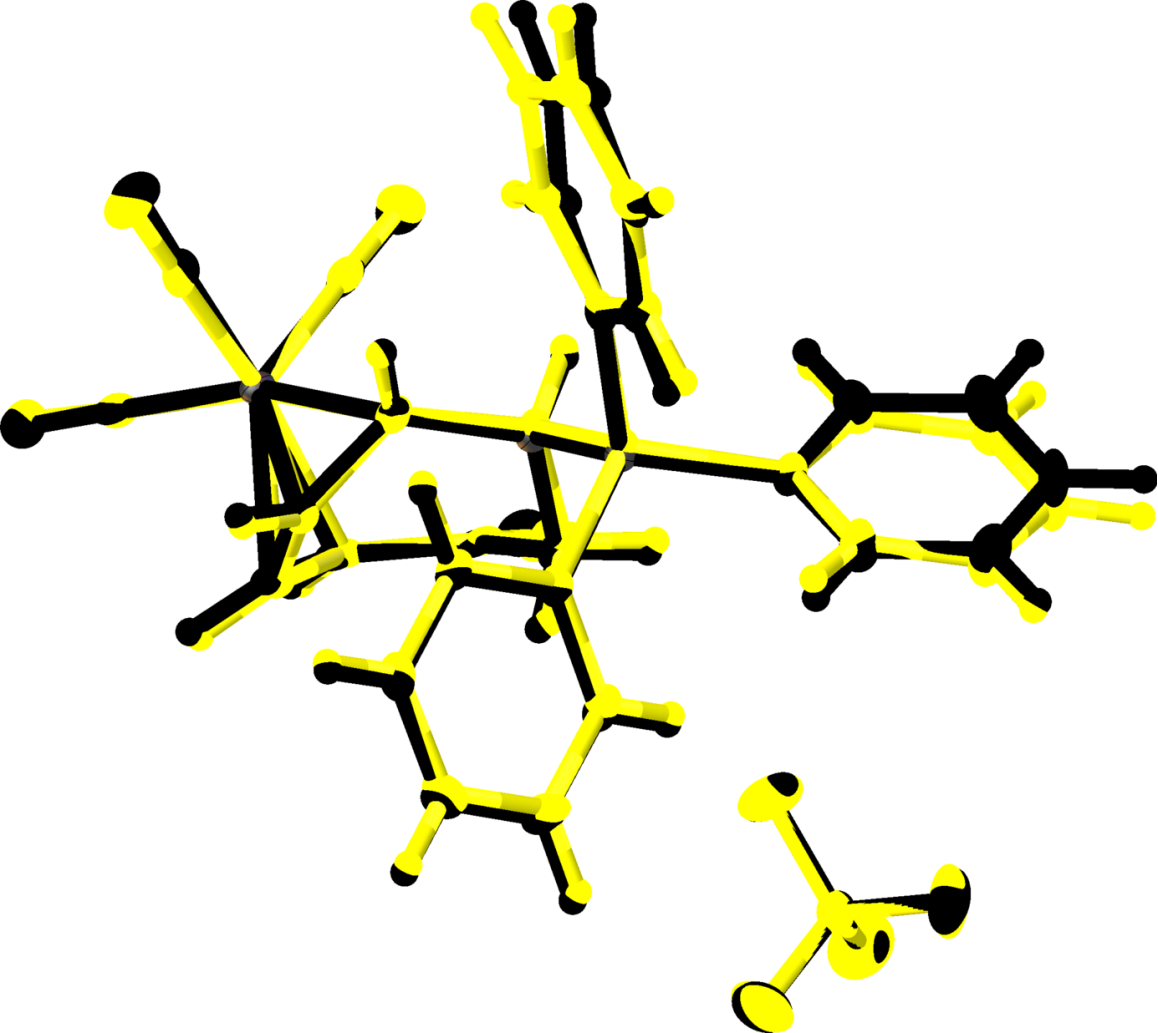
Mol­ecular overlay of between both cations constituting the symmetric unit of **III** demonstrating that the greatest disparity between them exists within the torsion angles of the the phenyl rings. The first tricarbon­yl[η^4^-6-*exo*-(tri­phenyl­phosphino)cyclo­hepta-2,4-dien-1-one]iron(0) tetra­fluor­r­borate is in black while the second is in yellow. Anisotropic displacement ellipsoids have been set to the 50% probability level.

The *Z*′ > 1 nature of the structural model for **III** suggests the presence of structural differences between mol­ecules within the asymmetric unit. Barring differences in the thermal parameters for the various atoms within the independent components, overlaying the tricarbon­yl[η^4^-6-*exo*-(tri­phenyl­phosphino)cyclo­hepta-2,4-dien-1-one]iron(0) cations and [BF_4_]^−^ anions showed that the anions had better alignment while differences in the some of the constituent torsion angles within phenyl rings from the tricarbon­yl[η^4^-6-*exo*-(tri­phenyl­phosphino)cyclo­hepta-2,4-dien-1-one]iron(0) cations were more pronounced visually (Fig. 6 ▸). Table 3 ▸ summarizes the torsion angles from the phenyl rings of the tricarbon­yl[η^4^-6-*exo*-(tri­phenyl­phosphino)cyclo­hepta-2,4-dien-1-one]iron(0) cations.

## 4. Database survey

The structure of this report is not found in the Cambridge Structural Database (CSD version 5.43; Groom *et al.*, 2016 ▸). To date, the structures of six tricarbon­yl(η^4^-tropone derivative)iron(0) compounds have been reported. In addition to the structure of compound **I** (Dodge, 1964 ▸), three of the remaining reports have one additional substituent in the 6-position, H (Sotokawa *et al.*, 1987 ▸), *t*-Bu (Coquerel *et al.*, 2002 ▸) and morpholi-4-yl (Huang *et al.*, 2018 ▸). From the various reports, comparison of their structural features suggested that the presence of the formally cationic phospho­rous had minimal impact on the bond lengths.

## 5. Synthesis and crystallization

All chemicals were purchased from commercial vendors and used as is. Compounds **I** and **II** were prepared according to literature procedures (Huang *et al.*, 2019 ▸). NMR spectra were obtained in *d*_3_-aceto­nitrile using a Bruker Avance III HD 400 FT-NMR spectrometer. The synthesis was performed using standard Schlenk conditions as outlined in Fig. 1 ▸, but all subsequent manipulations of the product were conducted in air. Compound **II** (0.0054 g, 0.016 mmol) and tri­phenyl­phosphine (0.0043 g, 0.016 mmol) were added to a 50 mL round-bottom flask along with a stir bar. Methyl­ene chloride (12 mL) was added, and the reaction mixture was stirred at room temperature for 30 minutes. A color change from pastel yellow to a darker yellow was observed. The solution was reduced *in vacuo* to approximately 5 mL and the resulting solution was layered with diethyl ether (7 mL) before being placed in the freezer for 48 h. The sample formed a pastel yellow solid and was filtered *via* cannula. The solid was dried *in vacuo* to give the desired product (0.0085 g, 88% yield). Crystals were grown by slow vapor diffusion of diethyl ether at room temperature into a solution of the compound in methyl­ene chloride. ^1^H NMR (400 MHz, CD_3_CN): *δ* 7.87 (*m*, 9H, H_*meta*_, H_*para*_), 7.75 (*m*, 6H, H_*ortho*_), 5.80 (*t*, *J* = 6.2 Hz, 1H, H4), 5.20 (*t*, *J* = 7.0 Hz, 1H, H1), 4.86 (*td*, *J* = 12.7, 4.7 Hz, 1H, H7), 3.21 (*dd*, *J* = 13.0, 7.5 Hz, 1H, H3), 3.16 (*d*, *J* = 6.6 Hz, 1H, H2), 2.18 (*m*, 1H, H6*A*/*B*), 1.99 (*q*, *J* = 12.2 Hz, 1H, H6*A*/*B*); ^31^P{^1^H} NMR (162 MHz, CD_3_CN): *δ* 23.3 (*s*); ^13^C{^1^H} NMR (100 MHz, CD_3_CN): *δ* 207.9 (*s*, No DEPT, C8–10), 202.6 (*d*, *J* = 15.4 Hz, No DEPT, C5), 135.9 (*d*, *J* = 3.2 Hz, DEPT +, C_*para*_), 134.8 (*d*, *J* = 9.5 Hz, DEPT +, C_*meta*_), 131.1 (*d*, *J* = 12.7 Hz, DEPT +, C_*ortho*_), 117.2 (*d*, *J* = 81.6 Hz, No DEPT, C_*ipso*_), 94.6 (*s*, DEPT +, C4), 89.8 (*s*, DEPT +, C1), 56.7 (*s*, DEPT +, C2), 49.5 (*d*, *J* = 7.4 Hz, DEPT +, C3), 41.2 (*d*, *J* = 31.8 Hz, DEPT +, C7), 37.2 (*s*, DEPT –, C6). Peaks were assigned using COSY, HMBC and HSQC NMR spectra. Protons of the tropone ring are labeled by the number of the carbon atom to which they are bonded. IR (cm^−1^, CH_3_CN): 2059 (*m*, Fe—C≡O), 2014 (*m*, Fe—C≡O), 1966 (*vs*, Fe—C≡O), 1710 (*m*, C=O), 1609 (*m*, C=C).

## 6. Refinement

Crystal data, data collection and structure refinement details are summarized in Table 4 ▸. All non-hydrogen atoms were refined anisotropically. H atoms bound to carbon were positioned geometrically and constrained to ride on their parent atoms. *U*_iso_(H) values were set to a multiple of *U*_eq_(C) with 1.2 times all CH and CH_2_ groups.

## Supplementary Material

Crystal structure: contains datablock(s) I. DOI: 10.1107/S2056989024005747/vu2002sup1.cif

Structure factors: contains datablock(s) I. DOI: 10.1107/S2056989024005747/vu2002Isup2.hkl

CCDC reference: 2362680

Additional supporting information:  crystallographic information; 3D view; checkCIF report

## Figures and Tables

**Figure 4:**
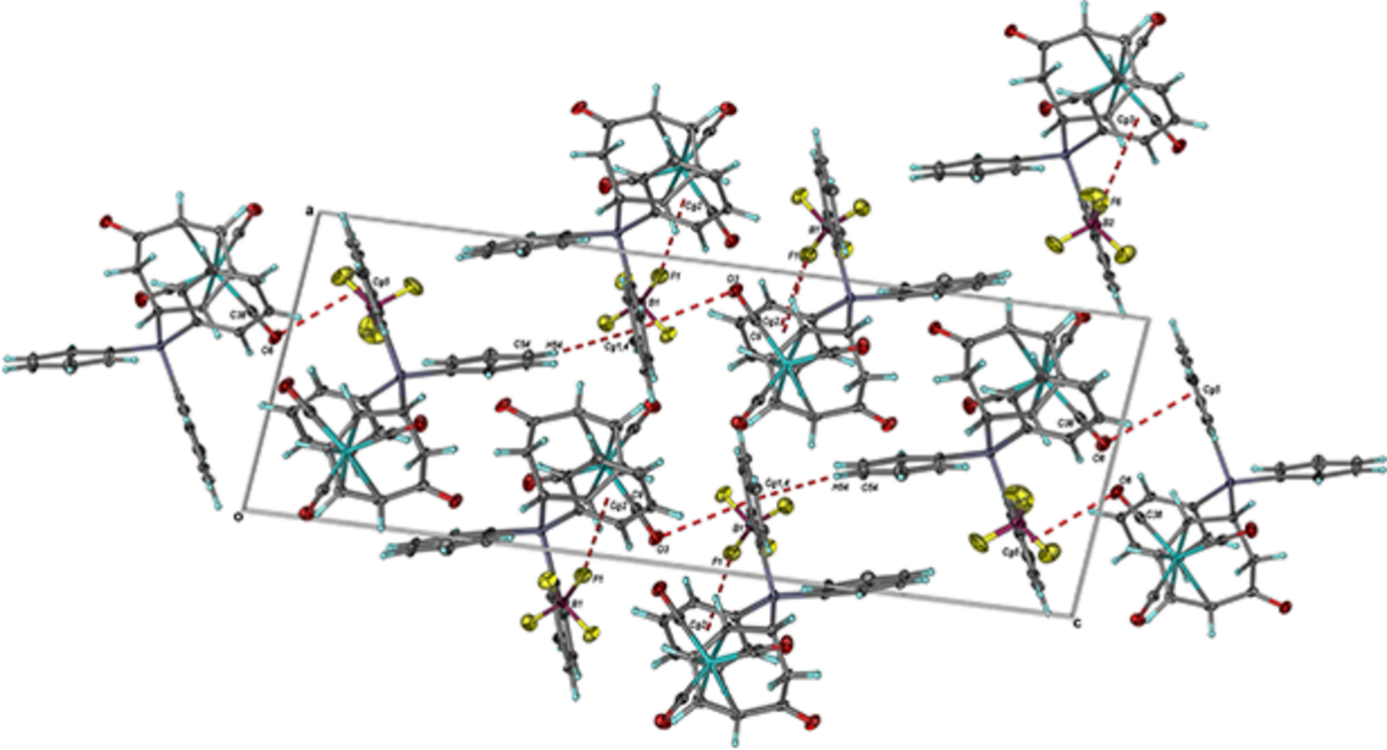
Projection of the C—H⋯π and Y—*X*⋯π (*Y* = B, C; *X* = F, O) inter­actions in the *ac* plane of **III**. Their combination with the hydrogen bonds yields a three-dimensional extended network in the solid state. Anisotropic displacement ellipsoids have been set to the 50% probability level (C_g_ = ring centroids).

**Table 1:** Hydrogen-bond geometry (Å, °)

*D*—H⋯*A*	*D*—H	H⋯*A*	*D*⋯*A*	*D*—H⋯*A*
C32—H32⋯F5	1.00	2.37	3.198 (3)	140
C43—H43⋯F6^i^	0.95	2.33	3.240 (3)	160
C44—H44⋯O8^ii^	0.95	2.56	3.467 (3)	159
C46—H46⋯F7^iii^	0.95	2.40	3.151 (3)	136
C49—H49⋯O7^iv^	0.95	2.60	3.339 (3)	135
C50—H50⋯F6^iv^	0.95	2.53	3.471 (3)	169
C52—H52⋯O1	0.95	2.37	3.286 (3)	161
C53—H53⋯F4^iii^	0.95	2.66	3.500 (3)	148
C56—H56⋯F6^iv^	0.95	2.48	3.362 (3)	155
C2—H2⋯F3^v^	1.00	2.50	3.493 (3)	170
C4—H4⋯F4^iii^	1.00	2.42	3.393 (3)	165
C7—H7⋯O5	1.00	2.34	3.192 (3)	143
C13—H13⋯F1^v^	0.95	2.41	3.232 (3)	144
C19—H19⋯O2^vi^	0.95	2.51	3.393 (3)	154
C28—H28⋯O5	0.95	2.41	3.319 (4)	161
C57*A*—H57*A*⋯F4	0.99	2.47	3.276 (4)	139

Symmetry codes: (i) 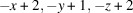; (ii) 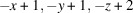; (iii) 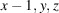; (iv) 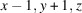; (v) 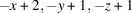; (vi) 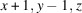.

**Table 2:** Phenyl ring torsion angles (°)

Cation 1		Cation 2	
Torsion angle	Value	Torsion angle	Value
Ring 1			
P1—C11—C16—C15	175.9 (2)	P2—C39—C40—C41	−176.5 (2)
C11—C16—C15—C14	−0.7 (4)	C39—C40—C41—C42	1.0 (4)
C16—C15—C14—C13	−0.7 (4)	C40—C41—C42—C43	1.2 (4)
C15—C14—C13—C12	1.2 (4)	C41—C42—C43—C44	−2.2 (4)
C14—C13—C12—C11	−0.2 (4)	C42—C43—C44—C39	0.9 (4)
C13—C12—C11—P1	−175.4 (2)	C43—C44—C39—P2	175.6 (2)
			
Ring 2			
P1—C17—C18—C19	−175.5 (2)	P2—C45—C50—C49	178.8 (2)
C17—C18—C19—C20	−0.1 (4)	C45—C50—C49—C48	0.8 (4)
C18—C19—C20—C21	0.4 (4)	C50—C49—C48—C47	−0.1 (4)
C19—C20—C21—C22	−0.2 (5)	C49—C48—C47—C46	−1.5 (4)
C20—C21—C22—C17	−1.2 (4)	C48—C47—C46—C45	2.3 (4)
C21—C22—C17—P1	176.0 (2)	C47—C46—C45—P2	179.7 (2)
			
Ring 3			
P1—C23—C28—C27	−170.3 (2)	P2—C51—C52—C53	176.9 (2)
C23—C28—C27—C26	−1.1 (4)	C51—C52—C53—C54	0.1 (4)
C28—C27—C26—C25	1.1 (4)	C52—C53—C54—C55	−0.5 (4)
C27—C26—C25—C24	0.1 (5)	C53—C54—C55—C56	−0.1 (5)
C26—C25—C24—C23	−1.3 (5)	C54—C55—C56—C51	1.2 (5)
C25—C24—C23—P1	171.4 (2)	C55—C56—C51—P2	−177.7 (2)

**Table 3:** *X*—*Y*⋯π inter­actions (Å,°) *Cg*1–*Cg*5 are the centroids of the C11–C16, C17–C22, C39–C44 and C45–C50 rings, respectively.

*X*—*Y*⋯*Cg*	*Y*⋯*Cg*	*X*⋯*Cg*	*X*—H⋯*Cg*
C54—H54⋯*Cg*2^i^	2.99	3.929 (3)	171
B1—F1⋯*Cg*1^ii^	3.429 (2)	4.790 (3)	165.56 (17)
B2—F6⋯*Cg*3^iii^	3.653 (2)	4.913 (3)	150.99 (17)
C9—O3⋯*Cg*2^iv^	3.393 (2)	3.858 (3)	105.37 (19)
C38—08⋯*Cg*4^v^	3.467 (2)	3.925 (3)	105.10 (18)

Symmetry codes: (i) −1 + *x*, 1 + *y*, *z*; (ii) *x*, 1 + *y*, *z*; (iii) 1 + *x*, −1 + *y*, *z*; (iv) 2 − *x*, 1 − *y*, 1 − *z*; (v) 1 − *x*, 1 − *y*, 2 − *z*.

**Table 4:** Experimental details

Crystal data	
Chemical formula	[Fe(C_28_H_22_O_4_)(CO)_3_]BF_4_·0.5CH_2_Cl_2_
*M*_r_	638.57
Crystal system, space group	Triclinic, *P* 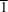
Temperature (K)	100
*a*, *b*, *c* (Å)	9.9343 (2), 10.9767 (3), 26.4168 (6)
α, β, γ (°)	86.993 (2), 82.468 (2), 77.300 (2)
*V* (Å^3^)	2785.09 (12)
*Z*	4
Radiation type	Mo *K*α
μ (mm^−1^)	0.76
Crystal size (mm)	0.3 × 0.14 × 0.08

Data collection	
Diffractometer	Rigaku Oxford Diffraction XtaLAB Mini II
Absorption correction	Analytical (*CrysAlis PRO*; Rigaku OD, 2023 ▸)
*T*_min_, *T*_max_	0.841, 0.969
No. of measured, independent and observed [*I* > 2σ(*I*)] reflections	59046, 9882, 7785
*R*_int_	0.054
(sin θ/λ)_max_ (Å^−1^)	0.597

Refinement	
*R*[*F*^2^ > 2σ(*F*^2^)], *wR*(*F*^2^), *S*	0.041, 0.103, 1.03
No. of reflections	9882
No. of parameters	730
No. of restraints	9
H-atom treatment	H-atom parameters constrained
Δρ_max_, Δρ_min_ (e Å^−3^)	0.54, −0.43

Computer programs: *CrysAlis PRO* (Rigaku OD, 2023 ▸), *SHELXT* (Sheldrick, 2015*a* ▸), *SHELXL2016/6* (Sheldrick, 2015*b* ▸) and *OLEX2* (Dolomanov *et al.*, 2009 ▸).

## supplementary crystallographic information

### Tricarbonyl[η4-6-exo-(triphenylphosphino)cyclohepta-2,4-dien-1-one]\ iron(0) tetrafluoroborate dichloromethane hemisolvate Crystal data

**Table:**

[Fe(C_28_H_22_O_4_)(CO)_3_]BF_4_·0.5CH_2_Cl_2_	*Z* = 4
*M**_r_* = 638.57	*F*(000) = 1300
Triclinic, *P*1	*D*_x_ = 1.523 Mg m^−^^3^
*a* = 9.9343 (2) Å	Mo *K*α radiation, λ = 0.71073 Å
*b* = 10.9767 (3) Å	Cell parameters from 14608 reflections
*c* = 26.4168 (6) Å	θ = 2.3–25.9°
α = 86.993 (2)°	µ = 0.76 mm^−^^1^
β = 82.468 (2)°	*T* = 100 K
γ = 77.300 (2)°	Block, yellow
*V* = 2785.09 (12) Å^3^	0.3 × 0.14 × 0.08 mm

### Tricarbonyl[η4-6-exo-(triphenylphosphino)cyclohepta-2,4-dien-1-one]\ iron(0) tetrafluoroborate dichloromethane hemisolvate Data collection

**Table:**

Rigaku Oxford Diffraction XtaLAB Mini II diffractometer	9882 independent reflections
Radiation source: fine-focus sealed X-ray tube, Rigaku (Mo) X-ray Source	7785 reflections with *I* > 2σ(*I*)
Graphite monochromator	*R*_int_ = 0.054
Detector resolution: 10.0000 pixels mm^-1^	θ_max_ = 25.1°, θ_min_ = 2.0°
ω scans	*h* = −11→11
Absorption correction: analytical (CrysAlisPro; Rigaku OD, 2023)	*k* = −13→13
*T*_min_ = 0.841, *T*_max_ = 0.969	*l* = −31→31
59046 measured reflections	

### Tricarbonyl[η4-6-exo-(triphenylphosphino)cyclohepta-2,4-dien-1-one]\ iron(0) tetrafluoroborate dichloromethane hemisolvate Refinement

**Table:**

Refinement on *F*^2^	Primary atom site location: dual
Least-squares matrix: full	Hydrogen site location: inferred from neighbouring sites
*R*[*F*^2^ > 2σ(*F*^2^)] = 0.041	H-atom parameters constrained
*wR*(*F*^2^) = 0.103	*w* = 1/[σ^2^(*F*_o_^2^) + (0.0554*P*)^2^ + 0.9998*P*] where *P* = (*F*_o_^2^ + 2*F*_c_^2^)/3
*S* = 1.03	(Δ/σ)_max_ = 0.001
9882 reflections	Δρ_max_ = 0.54 e Å^−^^3^
730 parameters	Δρ_min_ = −0.43 e Å^−^^3^
9 restraints	

### Tricarbonyl[η4-6-exo-(triphenylphosphino)cyclohepta-2,4-dien-1-one]\ iron(0) tetrafluoroborate dichloromethane hemisolvate Special details

**Table:**

Geometry. All esds (except the esd in the dihedral angle between two l.s. planes) are estimated using the full covariance matrix. The cell esds are taken into account individually in the estimation of esds in distances, angles and torsion angles; correlations between esds in cell parameters are only used when they are defined by crystal symmetry. An approximate (isotropic) treatment of cell esds is used for estimating esds involving l.s. planes.

### Tricarbonyl[η4-6-exo-(triphenylphosphino)cyclohepta-2,4-dien-1-one]\ iron(0) tetrafluoroborate dichloromethane hemisolvate Fractional atomic coordinates and isotropic or equivalent isotropic displacement parameters (Å^2^)

**Table:**

	*x*	*y*	*z*	*U*_iso_*/*U*_eq_	
P2	0.49890 (7)	0.80234 (6)	0.85505 (2)	0.01236 (16)	
Fe2	0.76338 (4)	0.40071 (4)	0.89551 (2)	0.01306 (11)	
O5	0.8797 (2)	0.49399 (18)	0.75394 (7)	0.0214 (5)	
O6	0.6413 (2)	0.28814 (19)	0.81779 (7)	0.0266 (5)	
O7	0.9804 (2)	0.18494 (18)	0.92305 (7)	0.0231 (5)	
O8	0.5743 (2)	0.3482 (2)	0.98587 (7)	0.0285 (5)	
C29	0.6417 (3)	0.5823 (2)	0.89315 (10)	0.0145 (6)	
H29	0.556816	0.593076	0.918838	0.017*	
C30	0.7655 (3)	0.5796 (2)	0.91661 (10)	0.0149 (6)	
H30	0.756979	0.598566	0.953652	0.018*	
C31	0.8961 (3)	0.5230 (2)	0.89111 (10)	0.0159 (6)	
H31	0.977935	0.502297	0.910803	0.019*	
C32	0.9086 (3)	0.4713 (3)	0.84151 (10)	0.0160 (6)	
H32	0.997745	0.409752	0.832129	0.019*	
C33	0.8442 (3)	0.5353 (3)	0.79735 (10)	0.0155 (6)	
C34	0.7409 (3)	0.6599 (2)	0.80507 (10)	0.0165 (6)	
H34A	0.713284	0.694001	0.771616	0.020*	
H34B	0.786063	0.719880	0.819411	0.020*	
C35	0.6098 (3)	0.6455 (2)	0.84144 (9)	0.0136 (6)	
H35	0.556541	0.595660	0.824317	0.016*	
C36	0.6867 (3)	0.3314 (3)	0.84864 (10)	0.0175 (6)	
C37	0.8977 (3)	0.2693 (3)	0.91181 (10)	0.0173 (6)	
C38	0.6482 (3)	0.3682 (3)	0.95096 (10)	0.0173 (6)	
C39	0.5932 (3)	0.8834 (2)	0.89063 (9)	0.0122 (6)	
C40	0.6901 (3)	0.9485 (2)	0.86506 (10)	0.0166 (6)	
H40	0.702505	0.953906	0.828798	0.020*	
C41	0.7676 (3)	1.0048 (3)	0.89304 (10)	0.0192 (6)	
H41	0.832112	1.049971	0.875878	0.023*	
C42	0.7511 (3)	0.9952 (3)	0.94643 (11)	0.0200 (7)	
H42	0.803154	1.035219	0.965383	0.024*	
C43	0.6592 (3)	0.9277 (3)	0.97171 (10)	0.0157 (6)	
H43	0.650592	0.919132	1.007893	0.019*	
C44	0.5792 (3)	0.8722 (2)	0.94406 (10)	0.0139 (6)	
H44	0.515241	0.826836	0.961476	0.017*	
C45	0.3386 (3)	0.7855 (2)	0.89292 (10)	0.0134 (6)	
C46	0.3015 (3)	0.6696 (3)	0.90128 (10)	0.0169 (6)	
H46	0.361384	0.596876	0.886685	0.020*	
C47	0.1768 (3)	0.6603 (3)	0.93104 (11)	0.0205 (7)	
H47	0.153518	0.581082	0.937688	0.025*	
C48	0.0867 (3)	0.7676 (3)	0.95092 (11)	0.0201 (7)	
H48	0.000884	0.761797	0.970466	0.024*	
C49	0.1227 (3)	0.8837 (3)	0.94208 (10)	0.0183 (6)	
H49	0.060856	0.956466	0.955709	0.022*	
C50	0.2479 (3)	0.8940 (3)	0.91362 (10)	0.0160 (6)	
H50	0.272247	0.973078	0.908149	0.019*	
C51	0.4586 (3)	0.8938 (2)	0.79783 (9)	0.0142 (6)	
C52	0.4425 (3)	0.8367 (3)	0.75349 (10)	0.0169 (6)	
H52	0.456999	0.748360	0.752392	0.020*	
C53	0.4047 (3)	0.9112 (3)	0.71075 (10)	0.0218 (7)	
H53	0.393439	0.873265	0.680526	0.026*	
C54	0.3839 (3)	1.0399 (3)	0.71241 (11)	0.0262 (7)	
H54	0.359061	1.089691	0.683175	0.031*	
C55	0.3990 (3)	1.0971 (3)	0.75671 (11)	0.0268 (7)	
H55	0.384692	1.185501	0.757477	0.032*	
C56	0.4353 (3)	1.0245 (3)	0.79987 (10)	0.0199 (7)	
H56	0.444019	1.063075	0.830287	0.024*	
P1	0.94285 (7)	0.20756 (6)	0.64553 (2)	0.01226 (16)	
O1	0.5518 (2)	0.53885 (18)	0.72809 (7)	0.0245 (5)	
Fe1	0.70408 (4)	0.60845 (4)	0.59115 (2)	0.01357 (11)	
C1	0.8213 (3)	0.4256 (2)	0.60006 (9)	0.0126 (6)	
H1	0.913470	0.411913	0.578387	0.015*	
C2	0.7095 (3)	0.4278 (2)	0.57052 (10)	0.0160 (6)	
H2	0.731133	0.405510	0.533685	0.019*	
O2	0.4934 (2)	0.81613 (18)	0.55330 (8)	0.0254 (5)	
C3	0.5726 (3)	0.4871 (3)	0.58972 (11)	0.0185 (6)	
H3	0.499787	0.506083	0.565967	0.022*	
O3	0.9219 (2)	0.6536 (2)	0.50941 (8)	0.0273 (5)	
C4	0.5435 (3)	0.5435 (3)	0.63893 (10)	0.0174 (6)	
H4	0.453410	0.606242	0.643897	0.021*	
O4	0.7946 (2)	0.73153 (19)	0.67411 (8)	0.0272 (5)	
C5	0.5929 (3)	0.4868 (3)	0.68714 (10)	0.0174 (6)	
C6	0.6918 (3)	0.3593 (3)	0.68483 (10)	0.0171 (6)	
H6A	0.649606	0.299488	0.668921	0.021*	
H6B	0.707112	0.328216	0.719929	0.021*	
C7	0.8327 (3)	0.3667 (2)	0.65366 (9)	0.0130 (6)	
H7	0.880827	0.416729	0.672986	0.016*	
C8	0.5753 (3)	0.7365 (3)	0.56836 (10)	0.0173 (6)	
C9	0.8363 (3)	0.6373 (3)	0.54088 (11)	0.0186 (6)	
C10	0.7618 (3)	0.6834 (3)	0.64182 (11)	0.0181 (6)	
C11	0.8592 (3)	0.1224 (2)	0.60744 (10)	0.0125 (6)	
C12	0.8886 (3)	0.1261 (2)	0.55398 (10)	0.0133 (6)	
H12	0.957399	0.168329	0.538081	0.016*	
C13	0.8167 (3)	0.0676 (2)	0.52443 (10)	0.0159 (6)	
H13	0.836029	0.070149	0.488247	0.019*	
C14	0.7169 (3)	0.0058 (3)	0.54771 (10)	0.0186 (6)	
H14	0.669280	−0.035317	0.527338	0.022*	
C15	0.6853 (3)	0.0030 (3)	0.60082 (10)	0.0177 (6)	
H15	0.615918	−0.039006	0.616404	0.021*	
C16	0.7559 (3)	0.0620 (2)	0.63085 (10)	0.0150 (6)	
H16	0.734259	0.061266	0.666957	0.018*	
C17	1.1110 (3)	0.2205 (2)	0.61364 (9)	0.0139 (6)	
C18	1.2036 (3)	0.1120 (3)	0.59409 (9)	0.0155 (6)	
H18	1.175348	0.034497	0.595495	0.019*	
C19	1.3374 (3)	0.1205 (3)	0.57264 (10)	0.0189 (7)	
H19	1.400826	0.047994	0.559412	0.023*	
C20	1.3790 (3)	0.2337 (3)	0.57043 (11)	0.0221 (7)	
H20	1.470133	0.238330	0.555546	0.026*	
C21	1.2875 (3)	0.3400 (3)	0.58992 (11)	0.0236 (7)	
H21	1.316252	0.417319	0.588198	0.028*	
C22	1.1544 (3)	0.3338 (3)	0.61191 (10)	0.0188 (6)	
H22	1.092670	0.406478	0.625785	0.023*	
C23	0.9730 (3)	0.1234 (3)	0.70496 (10)	0.0157 (6)	
C24	0.9921 (3)	−0.0070 (3)	0.70735 (11)	0.0226 (7)	
H24	0.976679	−0.050371	0.679188	0.027*	
C25	1.0334 (3)	−0.0724 (3)	0.75100 (12)	0.0298 (8)	
H25	1.044555	−0.160654	0.753021	0.036*	
C26	1.0588 (3)	−0.0089 (3)	0.79202 (11)	0.0272 (8)	
H26	1.087283	−0.054269	0.821806	0.033*	
C27	1.0425 (3)	0.1197 (3)	0.78950 (10)	0.0253 (7)	
H27	1.061477	0.162045	0.817316	0.030*	
C28	0.9983 (3)	0.1875 (3)	0.74612 (10)	0.0194 (6)	
H28	0.985532	0.275874	0.744555	0.023*	
B1	1.1632 (3)	0.7161 (3)	0.59857 (12)	0.0190 (7)	
F1	1.06555 (19)	0.81871 (17)	0.58349 (7)	0.0392 (5)	
F2	1.09598 (19)	0.62678 (16)	0.62366 (7)	0.0344 (5)	
F3	1.25338 (19)	0.66298 (17)	0.55619 (6)	0.0332 (5)	
F4	1.24188 (19)	0.75704 (18)	0.63210 (7)	0.0371 (5)	
B2	1.2845 (4)	0.2994 (3)	0.90769 (14)	0.0241 (8)	
F5	1.2026 (2)	0.3128 (2)	0.86803 (8)	0.0563 (6)	
F6	1.3542 (2)	0.17533 (15)	0.91095 (6)	0.0358 (5)	
F7	1.38046 (19)	0.37573 (18)	0.89795 (8)	0.0454 (5)	
F8	1.1993 (2)	0.33346 (18)	0.95371 (7)	0.0453 (5)	
Cl1A	1.25733 (10)	0.41520 (8)	0.72180 (3)	0.0411 (2)	
Cl2A	1.18892 (9)	0.61367 (8)	0.79693 (3)	0.0331 (2)	
C57A	1.1926 (4)	0.5766 (3)	0.73249 (11)	0.0316 (7)	
H57A	1.251916	0.624921	0.710549	0.038*	
H57B	1.097398	0.601362	0.722659	0.038*	

### Tricarbonyl[η4-6-exo-(triphenylphosphino)cyclohepta-2,4-dien-1-one]\ iron(0) tetrafluoroborate dichloromethane hemisolvate Atomic displacement parameters (Å^2^)

**Table:**

	*U*^11^	*U*^22^	*U*^33^	*U*^12^	*U*^13^	*U*^23^
P2	0.0146 (4)	0.0111 (4)	0.0105 (3)	−0.0010 (3)	−0.0010 (3)	−0.0018 (3)
Fe2	0.0128 (2)	0.0127 (2)	0.0132 (2)	−0.00214 (16)	−0.00068 (16)	−0.00078 (15)
O5	0.0223 (11)	0.0224 (12)	0.0170 (10)	−0.0010 (9)	0.0020 (9)	−0.0053 (8)
O6	0.0321 (13)	0.0305 (13)	0.0221 (11)	−0.0144 (10)	−0.0070 (10)	−0.0037 (10)
O7	0.0209 (11)	0.0180 (12)	0.0293 (11)	−0.0023 (10)	−0.0039 (9)	0.0023 (9)
O8	0.0297 (13)	0.0345 (14)	0.0203 (11)	−0.0111 (11)	0.0054 (10)	0.0043 (10)
C29	0.0149 (15)	0.0135 (15)	0.0123 (13)	−0.0005 (12)	0.0044 (11)	−0.0014 (11)
C30	0.0204 (15)	0.0107 (14)	0.0149 (13)	−0.0050 (12)	−0.0041 (12)	−0.0002 (11)
C31	0.0180 (15)	0.0121 (15)	0.0194 (14)	−0.0062 (12)	−0.0051 (12)	0.0019 (11)
C32	0.0106 (14)	0.0142 (15)	0.0230 (15)	−0.0035 (12)	0.0009 (12)	−0.0011 (12)
C33	0.0137 (14)	0.0141 (15)	0.0185 (14)	−0.0059 (12)	0.0034 (12)	−0.0006 (11)
C34	0.0195 (15)	0.0147 (15)	0.0128 (13)	−0.0009 (12)	0.0029 (11)	−0.0003 (11)
C35	0.0151 (14)	0.0111 (14)	0.0127 (13)	0.0011 (11)	−0.0014 (11)	−0.0019 (11)
C36	0.0163 (15)	0.0162 (16)	0.0181 (15)	−0.0027 (12)	0.0015 (12)	0.0036 (12)
C37	0.0190 (16)	0.0166 (16)	0.0171 (14)	−0.0066 (13)	0.0003 (12)	−0.0024 (12)
C38	0.0187 (16)	0.0125 (15)	0.0204 (15)	−0.0002 (12)	−0.0075 (13)	0.0006 (12)
C39	0.0112 (14)	0.0103 (14)	0.0140 (13)	0.0010 (11)	−0.0025 (11)	−0.0017 (10)
C40	0.0203 (15)	0.0146 (15)	0.0132 (13)	−0.0024 (12)	0.0013 (12)	−0.0007 (11)
C41	0.0157 (15)	0.0205 (16)	0.0219 (15)	−0.0079 (13)	0.0039 (12)	−0.0027 (12)
C42	0.0164 (15)	0.0222 (17)	0.0215 (15)	−0.0020 (13)	−0.0035 (12)	−0.0089 (12)
C43	0.0154 (15)	0.0174 (16)	0.0122 (13)	0.0011 (12)	−0.0009 (11)	−0.0029 (11)
C44	0.0146 (14)	0.0104 (14)	0.0149 (13)	−0.0006 (11)	0.0022 (11)	−0.0018 (11)
C45	0.0139 (14)	0.0151 (15)	0.0121 (13)	−0.0029 (12)	−0.0055 (11)	−0.0006 (11)
C46	0.0157 (15)	0.0161 (16)	0.0189 (14)	−0.0025 (12)	−0.0023 (12)	−0.0042 (12)
C47	0.0202 (16)	0.0203 (17)	0.0241 (15)	−0.0091 (13)	−0.0076 (13)	0.0030 (12)
C48	0.0120 (15)	0.0276 (18)	0.0216 (15)	−0.0054 (13)	−0.0027 (12)	−0.0015 (13)
C49	0.0144 (15)	0.0215 (17)	0.0170 (14)	0.0016 (12)	−0.0022 (12)	−0.0040 (12)
C50	0.0192 (15)	0.0127 (15)	0.0164 (14)	−0.0019 (12)	−0.0055 (12)	−0.0021 (11)
C51	0.0151 (14)	0.0132 (15)	0.0126 (13)	−0.0005 (12)	−0.0003 (11)	−0.0002 (11)
C52	0.0161 (15)	0.0173 (16)	0.0174 (14)	−0.0041 (12)	0.0000 (12)	−0.0042 (12)
C53	0.0190 (16)	0.0320 (19)	0.0136 (14)	−0.0036 (14)	−0.0025 (12)	−0.0015 (13)
C54	0.0269 (18)	0.033 (2)	0.0160 (15)	−0.0023 (15)	−0.0049 (13)	0.0083 (13)
C55	0.0343 (19)	0.0172 (17)	0.0269 (17)	−0.0002 (14)	−0.0066 (14)	0.0023 (13)
C56	0.0286 (17)	0.0125 (15)	0.0175 (14)	−0.0005 (13)	−0.0050 (13)	−0.0004 (12)
P1	0.0142 (4)	0.0106 (4)	0.0114 (3)	−0.0015 (3)	−0.0006 (3)	−0.0023 (3)
O1	0.0242 (12)	0.0230 (12)	0.0231 (11)	−0.0030 (9)	0.0086 (9)	−0.0082 (9)
Fe1	0.0123 (2)	0.0118 (2)	0.0157 (2)	−0.00230 (16)	0.00080 (16)	−0.00029 (16)
C1	0.0148 (14)	0.0106 (14)	0.0109 (12)	−0.0016 (11)	0.0028 (11)	−0.0023 (10)
C2	0.0225 (16)	0.0093 (14)	0.0171 (14)	−0.0055 (12)	−0.0020 (12)	−0.0013 (11)
O2	0.0208 (12)	0.0184 (12)	0.0360 (12)	−0.0016 (10)	−0.0063 (10)	0.0042 (10)
C3	0.0164 (15)	0.0165 (16)	0.0246 (15)	−0.0080 (12)	−0.0049 (12)	0.0059 (12)
O3	0.0255 (12)	0.0338 (14)	0.0228 (11)	−0.0115 (10)	0.0045 (10)	0.0023 (9)
C4	0.0087 (14)	0.0159 (16)	0.0258 (15)	−0.0025 (12)	0.0033 (12)	0.0025 (12)
O4	0.0359 (13)	0.0264 (13)	0.0219 (11)	−0.0117 (10)	−0.0031 (10)	−0.0035 (10)
C5	0.0112 (14)	0.0169 (16)	0.0230 (15)	−0.0059 (12)	0.0066 (12)	0.0000 (12)
C6	0.0187 (15)	0.0176 (16)	0.0130 (13)	−0.0031 (12)	0.0047 (12)	−0.0009 (11)
C7	0.0158 (14)	0.0106 (14)	0.0122 (13)	−0.0021 (11)	0.0004 (11)	−0.0031 (11)
C8	0.0152 (15)	0.0172 (16)	0.0205 (15)	−0.0087 (13)	0.0038 (12)	−0.0022 (12)
C9	0.0209 (16)	0.0161 (16)	0.0191 (15)	−0.0027 (13)	−0.0057 (13)	−0.0001 (12)
C10	0.0178 (15)	0.0135 (15)	0.0193 (15)	−0.0012 (12)	0.0058 (12)	0.0038 (12)
C11	0.0132 (14)	0.0097 (14)	0.0139 (13)	−0.0005 (11)	−0.0018 (11)	−0.0017 (11)
C12	0.0123 (14)	0.0115 (15)	0.0148 (13)	−0.0017 (11)	0.0019 (11)	−0.0012 (11)
C13	0.0170 (15)	0.0168 (15)	0.0124 (13)	0.0002 (12)	−0.0016 (11)	−0.0026 (11)
C14	0.0190 (16)	0.0179 (16)	0.0205 (14)	−0.0052 (13)	−0.0044 (12)	−0.0058 (12)
C15	0.0163 (15)	0.0154 (15)	0.0209 (14)	−0.0053 (12)	0.0025 (12)	−0.0011 (12)
C16	0.0185 (15)	0.0134 (15)	0.0126 (13)	−0.0026 (12)	−0.0010 (11)	−0.0014 (11)
C17	0.0146 (14)	0.0155 (15)	0.0111 (13)	−0.0011 (12)	−0.0026 (11)	−0.0019 (11)
C18	0.0174 (15)	0.0162 (15)	0.0139 (13)	−0.0036 (12)	−0.0045 (11)	−0.0028 (11)
C19	0.0129 (15)	0.0249 (17)	0.0161 (14)	0.0047 (13)	−0.0049 (12)	−0.0039 (12)
C20	0.0118 (15)	0.0287 (18)	0.0256 (16)	−0.0050 (13)	−0.0007 (12)	−0.0007 (13)
C21	0.0170 (16)	0.0215 (17)	0.0341 (17)	−0.0074 (13)	−0.0035 (13)	−0.0022 (14)
C22	0.0169 (15)	0.0156 (16)	0.0242 (15)	−0.0025 (12)	−0.0038 (12)	−0.0055 (12)
C23	0.0139 (14)	0.0177 (15)	0.0126 (13)	0.0000 (12)	0.0023 (11)	0.0015 (11)
C24	0.0269 (17)	0.0185 (17)	0.0221 (15)	−0.0032 (13)	−0.0052 (13)	−0.0004 (12)
C25	0.0345 (19)	0.0215 (18)	0.0311 (18)	−0.0018 (15)	−0.0054 (15)	0.0066 (14)
C26	0.0226 (17)	0.038 (2)	0.0151 (15)	0.0037 (15)	−0.0015 (13)	0.0095 (14)
C27	0.0242 (17)	0.034 (2)	0.0129 (14)	0.0034 (14)	0.0005 (12)	−0.0068 (13)
C28	0.0185 (16)	0.0220 (17)	0.0156 (14)	−0.0003 (13)	−0.0004 (12)	−0.0040 (12)
B1	0.0183 (18)	0.0163 (18)	0.0203 (17)	0.0000 (14)	−0.0015 (14)	−0.0007 (14)
F1	0.0316 (11)	0.0366 (12)	0.0390 (11)	0.0082 (9)	0.0001 (9)	0.0159 (9)
F2	0.0425 (12)	0.0244 (10)	0.0363 (10)	−0.0137 (9)	0.0039 (9)	0.0046 (8)
F3	0.0360 (11)	0.0324 (11)	0.0274 (10)	−0.0036 (9)	0.0068 (8)	−0.0092 (8)
F4	0.0352 (11)	0.0458 (13)	0.0344 (10)	−0.0141 (9)	−0.0067 (9)	−0.0093 (9)
B2	0.0226 (19)	0.0179 (19)	0.0313 (19)	−0.0027 (15)	−0.0054 (16)	0.0039 (15)
F5	0.0337 (12)	0.0883 (18)	0.0516 (13)	−0.0204 (12)	−0.0224 (10)	0.0308 (12)
F6	0.0588 (13)	0.0175 (10)	0.0240 (9)	0.0039 (9)	−0.0005 (9)	0.0003 (7)
F7	0.0285 (11)	0.0290 (12)	0.0799 (16)	−0.0121 (9)	0.0014 (10)	−0.0064 (11)
F8	0.0466 (13)	0.0345 (12)	0.0496 (12)	−0.0045 (10)	0.0119 (10)	−0.0154 (10)
Cl1A	0.0475 (6)	0.0249 (5)	0.0523 (5)	−0.0028 (4)	−0.0173 (4)	−0.0064 (4)
Cl2A	0.0452 (5)	0.0323 (5)	0.0239 (4)	−0.0120 (4)	−0.0065 (4)	0.0026 (3)
C57A	0.041 (2)	0.0278 (15)	0.0252 (14)	−0.0055 (15)	−0.0040 (14)	0.0010 (13)

### Tricarbonyl[η4-6-exo-(triphenylphosphino)cyclohepta-2,4-dien-1-one]\ iron(0) tetrafluoroborate dichloromethane hemisolvate Geometric parameters (Å, º)

**Table:**

P2—C35	1.853 (3)	Fe1—C2	2.071 (3)
P2—C39	1.799 (3)	Fe1—C3	2.065 (3)
P2—C45	1.807 (3)	Fe1—C4	2.127 (3)
P2—C51	1.810 (3)	Fe1—C8	1.816 (3)
Fe2—C29	2.094 (3)	Fe1—C9	1.809 (3)
Fe2—C30	2.074 (3)	Fe1—C10	1.822 (3)
Fe2—C31	2.068 (3)	C1—H1	1.0000
Fe2—C32	2.134 (3)	C1—C2	1.434 (4)
Fe2—C36	1.815 (3)	C1—C7	1.533 (3)
Fe2—C37	1.812 (3)	C2—H2	1.0000
Fe2—C38	1.808 (3)	C2—C3	1.412 (4)
O5—C33	1.234 (3)	O2—C8	1.149 (3)
O6—C36	1.150 (3)	C3—H3	1.0000
O7—C37	1.149 (3)	C3—C4	1.436 (4)
O8—C38	1.145 (3)	O3—C9	1.145 (3)
C29—H29	1.0000	C4—H4	1.0000
C29—C30	1.442 (4)	C4—C5	1.482 (4)
C29—C35	1.538 (3)	O4—C10	1.144 (3)
C30—H30	1.0000	C5—C6	1.522 (4)
C30—C31	1.408 (4)	C6—H6A	0.9900
C31—H31	1.0000	C6—H6B	0.9900
C31—C32	1.435 (4)	C6—C7	1.544 (4)
C32—H32	1.0000	C7—H7	1.0000
C32—C33	1.475 (4)	C11—C12	1.404 (3)
C33—C34	1.524 (4)	C11—C16	1.402 (4)
C34—H34A	0.9900	C12—H12	0.9500
C34—H34B	0.9900	C12—C13	1.390 (4)
C34—C35	1.546 (4)	C13—H13	0.9500
C35—H35	1.0000	C13—C14	1.384 (4)
C39—C40	1.405 (4)	C14—H14	0.9500
C39—C44	1.401 (3)	C14—C15	1.397 (4)
C40—H40	0.9500	C15—H15	0.9500
C40—C41	1.388 (4)	C15—C16	1.393 (4)
C41—H41	0.9500	C16—H16	0.9500
C41—C42	1.399 (4)	C17—C18	1.411 (4)
C42—H42	0.9500	C17—C22	1.400 (4)
C42—C43	1.383 (4)	C18—H18	0.9500
C43—H43	0.9500	C18—C19	1.396 (4)
C43—C44	1.396 (4)	C19—H19	0.9500
C44—H44	0.9500	C19—C20	1.389 (4)
C45—C46	1.399 (4)	C20—H20	0.9500
C45—C50	1.413 (4)	C20—C21	1.388 (4)
C46—H46	0.9500	C21—H21	0.9500
C46—C47	1.399 (4)	C21—C22	1.388 (4)
C47—H47	0.9500	C22—H22	0.9500
C47—C48	1.393 (4)	C23—C24	1.402 (4)
C48—H48	0.9500	C23—C28	1.403 (4)
C48—C49	1.398 (4)	C24—H24	0.9500
C49—H49	0.9500	C24—C25	1.386 (4)
C49—C50	1.390 (4)	C25—H25	0.9500
C50—H50	0.9500	C25—C26	1.397 (4)
C51—C52	1.400 (4)	C26—H26	0.9500
C51—C56	1.405 (4)	C26—C27	1.384 (4)
C52—H52	0.9500	C27—H27	0.9500
C52—C53	1.401 (4)	C27—C28	1.400 (4)
C53—H53	0.9500	C28—H28	0.9500
C53—C54	1.384 (4)	B1—F1	1.394 (4)
C54—H54	0.9500	B1—F2	1.396 (4)
C54—C55	1.397 (4)	B1—F3	1.404 (3)
C55—H55	0.9500	B1—F4	1.406 (4)
C55—C56	1.397 (4)	B2—F5	1.392 (4)
C56—H56	0.9500	B2—F6	1.389 (4)
P1—C7	1.853 (3)	B2—F7	1.393 (4)
P1—C11	1.799 (3)	B2—F8	1.405 (4)
P1—C17	1.801 (3)	Cl1A—C57A	1.771 (3)
P1—C23	1.805 (3)	Cl2A—C57A	1.765 (3)
O1—C5	1.229 (3)	C57A—H57A	0.9900
Fe1—C1	2.106 (3)	C57A—H57B	0.9900
			
C39—P2—C35	107.22 (12)	C3—Fe1—C1	72.32 (11)
C39—P2—C45	109.80 (12)	C3—Fe1—C2	39.93 (11)
C39—P2—C51	108.45 (12)	C3—Fe1—C4	40.04 (10)
C45—P2—C35	109.27 (12)	C8—Fe1—C1	159.35 (11)
C45—P2—C51	109.02 (12)	C8—Fe1—C2	119.34 (12)
C51—P2—C35	113.05 (12)	C8—Fe1—C3	90.88 (12)
C29—Fe2—C32	85.14 (10)	C8—Fe1—C4	89.74 (11)
C30—Fe2—C29	40.48 (10)	C8—Fe1—C10	101.08 (12)
C30—Fe2—C32	72.09 (10)	C9—Fe1—C1	89.05 (11)
C31—Fe2—C29	72.52 (11)	C9—Fe1—C2	95.78 (12)
C31—Fe2—C30	39.75 (10)	C9—Fe1—C3	127.05 (12)
C31—Fe2—C32	39.90 (10)	C9—Fe1—C4	167.04 (12)
C36—Fe2—C29	98.70 (11)	C9—Fe1—C8	91.88 (12)
C36—Fe2—C30	136.74 (11)	C9—Fe1—C10	96.79 (12)
C36—Fe2—C31	134.13 (11)	C10—Fe1—C1	99.29 (11)
C36—Fe2—C32	95.62 (11)	C10—Fe1—C2	137.07 (11)
C37—Fe2—C29	160.40 (12)	C10—Fe1—C3	134.26 (11)
C37—Fe2—C30	120.23 (12)	C10—Fe1—C4	95.51 (11)
C37—Fe2—C31	92.41 (12)	Fe1—C1—H1	112.0
C37—Fe2—C32	91.19 (11)	C2—C1—Fe1	68.62 (15)
C37—Fe2—C36	100.82 (12)	C2—C1—H1	112.0
C38—Fe2—C29	88.42 (11)	C2—C1—C7	125.5 (2)
C38—Fe2—C30	95.26 (11)	C7—C1—Fe1	119.90 (17)
C38—Fe2—C31	126.49 (11)	C7—C1—H1	112.0
C38—Fe2—C32	166.34 (12)	Fe1—C2—H2	119.4
C38—Fe2—C36	97.26 (12)	C1—C2—Fe1	71.24 (15)
C38—Fe2—C37	90.87 (12)	C1—C2—H2	119.4
Fe2—C29—H29	111.9	C3—C2—Fe1	69.78 (15)
C30—C29—Fe2	69.04 (15)	C3—C2—C1	119.7 (2)
C30—C29—H29	111.9	C3—C2—H2	119.4
C30—C29—C35	125.5 (2)	Fe1—C3—H3	118.9
C35—C29—Fe2	119.85 (17)	C2—C3—Fe1	70.29 (15)
C35—C29—H29	111.9	C2—C3—H3	118.9
Fe2—C30—H30	119.5	C2—C3—C4	120.8 (2)
C29—C30—Fe2	70.48 (15)	C4—C3—Fe1	72.34 (16)
C29—C30—H30	119.5	C4—C3—H3	118.9
C31—C30—Fe2	69.88 (15)	Fe1—C4—H4	114.0
C31—C30—C29	119.4 (2)	C3—C4—Fe1	67.62 (15)
C31—C30—H30	119.5	C3—C4—H4	114.0
Fe2—C31—H31	118.8	C3—C4—C5	126.6 (2)
C30—C31—Fe2	70.37 (16)	C5—C4—Fe1	111.10 (18)
C30—C31—H31	118.8	C5—C4—H4	114.0
C30—C31—C32	121.2 (2)	O1—C5—C4	121.2 (3)
C32—C31—Fe2	72.50 (15)	O1—C5—C6	120.7 (3)
C32—C31—H31	118.8	C4—C5—C6	118.0 (2)
Fe2—C32—H32	114.1	C5—C6—H6A	109.4
C31—C32—Fe2	67.59 (15)	C5—C6—H6B	109.4
C31—C32—H32	114.1	C5—C6—C7	111.0 (2)
C31—C32—C33	125.5 (2)	H6A—C6—H6B	108.0
C33—C32—Fe2	112.39 (18)	C7—C6—H6A	109.4
C33—C32—H32	114.1	C7—C6—H6B	109.4
O5—C33—C32	121.1 (2)	P1—C7—H7	108.6
O5—C33—C34	119.6 (2)	C1—C7—P1	106.93 (17)
C32—C33—C34	119.1 (2)	C1—C7—C6	114.4 (2)
C33—C34—H34A	109.3	C1—C7—H7	108.6
C33—C34—H34B	109.3	C6—C7—P1	109.73 (18)
C33—C34—C35	111.5 (2)	C6—C7—H7	108.6
H34A—C34—H34B	108.0	O2—C8—Fe1	178.8 (3)
C35—C34—H34A	109.3	O3—C9—Fe1	178.6 (3)
C35—C34—H34B	109.3	O4—C10—Fe1	178.2 (3)
P2—C35—H35	108.9	C12—C11—P1	119.8 (2)
C29—C35—P2	107.10 (17)	C16—C11—P1	119.85 (19)
C29—C35—C34	113.7 (2)	C16—C11—C12	120.1 (2)
C29—C35—H35	108.9	C11—C12—H12	120.1
C34—C35—P2	109.12 (18)	C13—C12—C11	119.8 (2)
C34—C35—H35	108.9	C13—C12—H12	120.1
O6—C36—Fe2	177.8 (2)	C12—C13—H13	120.0
O7—C37—Fe2	178.2 (3)	C14—C13—C12	120.0 (2)
O8—C38—Fe2	179.3 (3)	C14—C13—H13	120.0
C40—C39—P2	120.3 (2)	C13—C14—H14	119.6
C44—C39—P2	119.8 (2)	C13—C14—C15	120.8 (3)
C44—C39—C40	119.7 (2)	C15—C14—H14	119.6
C39—C40—H40	120.2	C14—C15—H15	120.1
C41—C40—C39	119.7 (2)	C16—C15—C14	119.8 (3)
C41—C40—H40	120.2	C16—C15—H15	120.1
C40—C41—H41	119.9	C11—C16—H16	120.2
C40—C41—C42	120.3 (3)	C15—C16—C11	119.6 (2)
C42—C41—H41	119.9	C15—C16—H16	120.2
C41—C42—H42	119.9	C18—C17—P1	118.9 (2)
C43—C42—C41	120.3 (3)	C22—C17—P1	120.9 (2)
C43—C42—H42	119.9	C22—C17—C18	120.0 (2)
C42—C43—H43	120.0	C17—C18—H18	120.6
C42—C43—C44	120.0 (2)	C19—C18—C17	118.8 (3)
C44—C43—H43	120.0	C19—C18—H18	120.6
C39—C44—H44	120.0	C18—C19—H19	119.6
C43—C44—C39	120.1 (3)	C20—C19—C18	120.7 (3)
C43—C44—H44	120.0	C20—C19—H19	119.6
C46—C45—P2	122.0 (2)	C19—C20—H20	119.9
C46—C45—C50	119.8 (3)	C21—C20—C19	120.2 (3)
C50—C45—P2	118.2 (2)	C21—C20—H20	119.9
C45—C46—H46	119.9	C20—C21—H21	119.9
C47—C46—C45	120.3 (3)	C22—C21—C20	120.3 (3)
C47—C46—H46	119.9	C22—C21—H21	119.9
C46—C47—H47	120.1	C17—C22—H22	120.0
C48—C47—C46	119.8 (3)	C21—C22—C17	120.0 (3)
C48—C47—H47	120.1	C21—C22—H22	120.0
C47—C48—H48	120.0	C24—C23—P1	120.0 (2)
C47—C48—C49	119.9 (3)	C24—C23—C28	120.2 (3)
C49—C48—H48	120.0	C28—C23—P1	119.0 (2)
C48—C49—H49	119.6	C23—C24—H24	120.2
C50—C49—C48	120.9 (3)	C25—C24—C23	119.7 (3)
C50—C49—H49	119.6	C25—C24—H24	120.2
C45—C50—H50	120.4	C24—C25—H25	119.9
C49—C50—C45	119.2 (3)	C24—C25—C26	120.2 (3)
C49—C50—H50	120.4	C26—C25—H25	119.9
C52—C51—P2	121.0 (2)	C25—C26—H26	119.8
C52—C51—C56	120.5 (2)	C27—C26—C25	120.4 (3)
C56—C51—P2	118.3 (2)	C27—C26—H26	119.8
C51—C52—H52	120.3	C26—C27—H27	119.9
C51—C52—C53	119.3 (3)	C26—C27—C28	120.2 (3)
C53—C52—H52	120.3	C28—C27—H27	119.9
C52—C53—H53	119.9	C23—C28—H28	120.4
C54—C53—C52	120.2 (3)	C27—C28—C23	119.3 (3)
C54—C53—H53	119.9	C27—C28—H28	120.4
C53—C54—H54	119.7	F1—B1—F2	110.1 (3)
C53—C54—C55	120.6 (3)	F1—B1—F3	110.7 (2)
C55—C54—H54	119.7	F1—B1—F4	108.4 (3)
C54—C55—H55	120.0	F2—B1—F3	109.8 (2)
C56—C55—C54	120.1 (3)	F2—B1—F4	109.3 (2)
C56—C55—H55	120.0	F3—B1—F4	108.6 (2)
C51—C56—H56	120.4	F5—B2—F7	109.7 (3)
C55—C56—C51	119.3 (3)	F5—B2—F8	109.4 (3)
C55—C56—H56	120.4	F6—B2—F5	108.6 (3)
C11—P1—C7	107.74 (12)	F6—B2—F7	109.7 (3)
C11—P1—C17	110.56 (12)	F6—B2—F8	109.9 (3)
C11—P1—C23	109.76 (13)	F7—B2—F8	109.5 (3)
C17—P1—C7	108.54 (12)	Cl1A—C57A—H57A	109.2
C17—P1—C23	106.44 (12)	Cl1A—C57A—H57B	109.2
C23—P1—C7	113.81 (12)	Cl2A—C57A—Cl1A	112.05 (17)
C1—Fe1—C4	84.95 (10)	Cl2A—C57A—H57A	109.2
C2—Fe1—C1	40.14 (10)	Cl2A—C57A—H57B	109.2
C2—Fe1—C4	72.30 (11)	H57A—C57A—H57B	107.9
			
P2—C39—C40—C41	−176.5 (2)	P1—C11—C12—C13	−175.4 (2)
P2—C39—C44—C43	175.57 (19)	P1—C11—C16—C15	175.9 (2)
P2—C45—C46—C47	179.7 (2)	P1—C17—C18—C19	−175.46 (19)
P2—C45—C50—C49	178.8 (2)	P1—C17—C22—C21	176.0 (2)
P2—C51—C52—C53	176.9 (2)	P1—C23—C24—C25	171.4 (2)
P2—C51—C56—C55	−177.7 (2)	P1—C23—C28—C27	−170.3 (2)
Fe2—C29—C30—C31	51.8 (2)	O1—C5—C6—C7	−116.4 (3)
Fe2—C29—C35—P2	179.24 (13)	Fe1—C1—C2—C3	−52.1 (2)
Fe2—C29—C35—C34	−60.1 (3)	Fe1—C1—C7—P1	179.98 (13)
Fe2—C30—C31—C32	53.9 (2)	Fe1—C1—C7—C6	58.3 (3)
Fe2—C31—C32—C33	102.3 (3)	Fe1—C2—C3—C4	−53.9 (2)
Fe2—C32—C33—O5	−115.5 (2)	Fe1—C3—C4—C5	−100.3 (3)
Fe2—C32—C33—C34	69.6 (3)	Fe1—C4—C5—O1	109.3 (3)
O5—C33—C34—C35	121.2 (3)	Fe1—C4—C5—C6	−73.2 (3)
C29—C30—C31—Fe2	−52.0 (2)	C1—C2—C3—Fe1	52.8 (2)
C29—C30—C31—C32	1.9 (4)	C1—C2—C3—C4	−1.1 (4)
C30—C29—C35—P2	−96.3 (3)	C2—C1—C7—P1	96.1 (3)
C30—C29—C35—C34	24.3 (4)	C2—C1—C7—C6	−25.6 (4)
C30—C31—C32—Fe2	−53.0 (2)	C2—C3—C4—Fe1	53.0 (2)
C30—C31—C32—C33	49.3 (4)	C2—C3—C4—C5	−47.4 (4)
C31—C32—C33—O5	166.8 (3)	C3—C4—C5—O1	−173.5 (3)
C31—C32—C33—C34	−8.1 (4)	C3—C4—C5—C6	4.0 (4)
C32—C33—C34—C35	−63.8 (3)	C4—C5—C6—C7	66.0 (3)
C33—C34—C35—P2	173.40 (18)	C5—C6—C7—P1	−173.67 (18)
C33—C34—C35—C29	53.9 (3)	C5—C6—C7—C1	−53.5 (3)
C35—P2—C39—C40	85.3 (2)	C7—P1—C11—C12	89.3 (2)
C35—P2—C39—C44	−88.9 (2)	C7—P1—C11—C16	−85.0 (2)
C35—P2—C45—C46	−10.1 (3)	C7—P1—C17—C18	−167.5 (2)
C35—P2—C45—C50	171.1 (2)	C7—P1—C17—C22	17.9 (3)
C35—P2—C51—C52	33.2 (3)	C7—P1—C23—C24	149.2 (2)
C35—P2—C51—C56	−150.7 (2)	C7—P1—C23—C28	−40.5 (3)
C35—C29—C30—Fe2	−112.4 (2)	C7—C1—C2—Fe1	112.2 (2)
C35—C29—C30—C31	−60.7 (4)	C7—C1—C2—C3	60.1 (4)
C39—P2—C35—C29	57.4 (2)	C11—P1—C7—C1	−59.7 (2)
C39—P2—C35—C34	−66.1 (2)	C11—P1—C7—C6	64.9 (2)
C39—P2—C45—C46	−127.4 (2)	C11—P1—C17—C18	−49.5 (2)
C39—P2—C45—C50	53.8 (2)	C11—P1—C17—C22	135.9 (2)
C39—P2—C51—C52	151.9 (2)	C11—P1—C23—C24	28.4 (3)
C39—P2—C51—C56	−32.0 (3)	C11—P1—C23—C28	−161.3 (2)
C39—C40—C41—C42	1.0 (4)	C11—C12—C13—C14	−0.2 (4)
C40—C39—C44—C43	1.3 (4)	C12—C11—C16—C15	1.6 (4)
C40—C41—C42—C43	1.2 (4)	C12—C13—C14—C15	1.2 (4)
C41—C42—C43—C44	−2.1 (4)	C13—C14—C15—C16	−0.7 (4)
C42—C43—C44—C39	0.9 (4)	C14—C15—C16—C11	−0.7 (4)
C44—C39—C40—C41	−2.2 (4)	C16—C11—C12—C13	−1.2 (4)
C45—P2—C35—C29	−61.5 (2)	C17—P1—C7—C1	60.1 (2)
C45—P2—C35—C34	174.99 (17)	C17—P1—C7—C6	−175.37 (18)
C45—P2—C39—C40	−156.1 (2)	C17—P1—C11—C12	−29.1 (2)
C45—P2—C39—C44	29.7 (2)	C17—P1—C11—C16	156.6 (2)
C45—P2—C51—C52	−88.5 (2)	C17—P1—C23—C24	−91.3 (2)
C45—P2—C51—C56	87.5 (2)	C17—P1—C23—C28	79.0 (2)
C45—C46—C47—C48	2.3 (4)	C17—C18—C19—C20	−0.1 (4)
C46—C45—C50—C49	0.0 (4)	C18—C17—C22—C21	1.4 (4)
C46—C47—C48—C49	−1.5 (4)	C18—C19—C20—C21	0.4 (4)
C47—C48—C49—C50	−0.1 (4)	C19—C20—C21—C22	0.3 (4)
C48—C49—C50—C45	0.8 (4)	C20—C21—C22—C17	−1.2 (4)
C50—C45—C46—C47	−1.5 (4)	C22—C17—C18—C19	−0.8 (4)
C51—P2—C35—C29	176.90 (17)	C23—P1—C7—C1	178.39 (18)
C51—P2—C35—C34	53.4 (2)	C23—P1—C7—C6	−57.1 (2)
C51—P2—C39—C40	−37.1 (2)	C23—P1—C11—C12	−146.3 (2)
C51—P2—C39—C44	148.7 (2)	C23—P1—C11—C16	39.5 (2)
C51—P2—C45—C46	113.9 (2)	C23—P1—C17—C18	69.6 (2)
C51—P2—C45—C50	−64.9 (2)	C23—P1—C17—C22	−105.0 (2)
C51—C52—C53—C54	0.1 (4)	C23—C24—C25—C26	−1.3 (5)
C52—C51—C56—C55	−1.6 (4)	C24—C23—C28—C27	0.0 (4)
C52—C53—C54—C55	−0.5 (4)	C24—C25—C26—C27	0.1 (5)
C53—C54—C55—C56	−0.1 (5)	C25—C26—C27—C28	1.1 (4)
C54—C55—C56—C51	1.2 (5)	C26—C27—C28—C23	−1.1 (4)
C56—C51—C52—C53	1.0 (4)	C28—C23—C24—C25	1.2 (4)

### Tricarbonyl[η4-6-exo-(triphenylphosphino)cyclohepta-2,4-dien-1-one]\ iron(0) tetrafluoroborate dichloromethane hemisolvate Hydrogen-bond geometry (Å, º)

**Table:**

*D*—H···*A*	*D*—H	H···*A*	*D*···*A*	*D*—H···*A*
C32—H32···F5	1.00	2.37	3.198 (3)	140
C43—H43···F6^i^	0.95	2.33	3.240 (3)	160
C44—H44···O8^ii^	0.95	2.56	3.467 (3)	159
C46—H46···F7^iii^	0.95	2.40	3.151 (3)	136
C49—H49···O7^iv^	0.95	2.60	3.339 (3)	135
C50—H50···F6^iv^	0.95	2.53	3.471 (3)	169
C52—H52···O1	0.95	2.37	3.286 (3)	161
C53—H53···F4^iii^	0.95	2.66	3.500 (3)	148
C56—H56···F6^iv^	0.95	2.48	3.362 (3)	155
C2—H2···F3^v^	1.00	2.50	3.493 (3)	170
C4—H4···F4^iii^	1.00	2.42	3.393 (3)	165
C7—H7···O5	1.00	2.34	3.192 (3)	143
C13—H13···F1^v^	0.95	2.41	3.232 (3)	144
C19—H19···O2^vi^	0.95	2.51	3.393 (3)	154
C28—H28···O5	0.95	2.41	3.319 (4)	161
C57*A*—H57*A*···F4	0.99	2.47	3.276 (4)	139

Symmetry codes: (i) −*x*+2, −*y*+1, −*z*+2; (ii) −*x*+1, −*y*+1, −*z*+2; (iii) *x*−1, *y*, *z*; (iv) *x*−1, *y*+1, *z*; (v) −*x*+2, −*y*+1, −*z*+1; (vi) *x*+1, *y*−1, *z*.

## References

[bb1] Brown, D. A., Chawla, S. K., Glass, W. K. & Hussein, F. M. (1982). *Inorg. Chem.* **21**, 2726–2732.

[bb3] Coquerel, Y., Deprés, J.-P., Greene, A. E. & Philouze, C. (2002). *J. Organomet. Chem.* **659**, 176–185.

[bb4] Dodge, R. P. (1964). *J. Am. Chem. Soc.* **86**, 5429–5431.

[bb5] Dolomanov, O. V., Bourhis, L. J., Gildea, R. J., Howard, J. A. K. & Puschmann, H. (2009). *J. Appl. Cryst.* **42**, 339–341.

[bb6] Eisenstadt, A. (1975). *J. Organomet. Chem.* **97**, 443–451.

[bb7] Groom, C. R., Bruno, I. J., Lightfoot, M. P. & Ward, S. C. (2016). *Acta Cryst.* B**72**, 171–179. 10.1107/S2052520616003954 PMC4822653 27048719

[bb8] Huang, Z., Phelan, Z. K., Tritt, R. L., Valent, S. D. & Griffith, D. R. (2018). *Tetrahedron Lett.* **59**, 3432–3434.

[bb9] Huang, Z., Phelan, Z. K., Tritt, R. L., Valent, S. D., Guan, Z., He, Y., Weiss, P. S. & Griffith, D. R. (2019). *J. Vis. Exp.* **150**, e60050. 10.3791/60050 31449244

[bb10] Phelan, Z. K., Weiss, P. S., He, Y., Guan, Z., Thamattoor, D. M. & Griffith, D. R. (2020). *J. Org. Chem.* **85**, 2202–2212. 10.1021/acs.joc.9b02921 31904976

[bb2] Rigaku OD (2023). *CrysAlis PRO*. Rigaku Oxford Diffraction, Yarnton, England.

[bb11] Sheldrick, G. M. (2015*a*). *Acta Cryst.* A**71**, 3–8.

[bb12] Sheldrick, G. M. (2015*b*). *Acta Cryst.* C**71**, 3–8.

[bb13] Shoemaker, A. H. & Griffith, D. R. (2021). *Synthesis*, **53**, 65–78.

[bb14] Sotokawa, H., Tajiri, A., Morita, N., Kabuto, C., Hatano, M. & Asao, T. (1987). *Tetrahedron Lett.* **28**, 5873–5876.

[bb15] Spek, A. L. (2020). *Acta Cryst.* E**76**, 1–11. 10.1107/S2056989019016244 PMC6944088 31921444

[bb16] Stone, K. L., Kissel, D. S., Shaner, S. E., Grice, K. A. & Van Opstal, M. T. (2020). *ACS Symp. Ser.* **1371**, 35–55.

